# Lipid Anchoring of Archaeosortase Substrates and Midcell Growth in Haloarchaea

**DOI:** 10.1128/mBio.00349-20

**Published:** 2020-03-24

**Authors:** Mohd Farid Abdul-Halim, Stefan Schulze, Anthony DiLucido, Friedhelm Pfeiffer, Alexandre Wilson Bisson Filho, Mechthild Pohlschroder

**Affiliations:** a Department of Biology, University of Pennsylvania, Philadelphia, Pennsylvania, USA; b Computational Biology Group, Max Planck Institute of Biochemistry, Martinsried, Germany; c Department of Biology, Rosenstiel Basic Medical Science Research Center, Brandeis University, Waltham, Massachusetts, USA; University of Freiburg; University of Georgia

**Keywords:** archaea, *Haloferax volcanii*, S-layer, archaeosortase, cell division, cell elongation, cell shape, cell surface, haloarchaea, lipid anchoring

## Abstract

The archaeal cytoplasmic membrane provides an anchor for many surface proteins. Recently, a novel membrane anchoring mechanism involving a peptidase, archaeosortase A (ArtA), and C-terminal lipid attachment of surface proteins was identified in the model archaeon Haloferax volcanii. ArtA is required for optimal cell growth and morphogenesis, and the S-layer glycoprotein (SLG), the sole component of the H. volcanii cell wall, is one of the targets for this anchoring mechanism. However, how exactly ArtA function and regulation control cell growth and morphogenesis is still elusive. Here, we report that archaeal homologs to the bacterial phosphatidylserine synthase (PssA) and phosphatidylserine decarboxylase (PssD) are involved in ArtA-dependent protein maturation. Haloferax volcanii strains lacking either HvPssA or HvPssD exhibited motility, growth, and morphological phenotypes similar to those of an Δ*artA* mutant. Moreover, we showed a loss of covalent lipid attachment to SLG in the Δ*hvpssA* mutant and that proteolytic cleavage of the ArtA substrate HVO_0405 was blocked in the Δ*hvpssA* and Δ*hvpssD* mutant strains. Strikingly, ArtA, HvPssA, and HvPssD green fluorescent protein (GFP) fusions colocalized to the midcell position of H. volcanii cells, strongly supporting that they are involved in the same pathway. Finally, we have shown that the SLG is also recruited to the midcell before being secreted and lipid anchored at the cell outer surface. Collectively, our data suggest that haloarchaea use the midcell as the main surface processing hot spot for cell elongation, division, and shape determination.

## Abstract (precis)

The subcellular organization of biochemical processes in space and time is still one of the most mysterious topics in archaeal cell biology. Despite the fact that haloarchaea largely rely on covalent lipid anchoring to coat the cell envelope, little is known about how cells coordinate *de novo* synthesis and about the insertion of this proteinaceous layer throughout the cell cycle. Here, we report the identification of two novel contributors to ArtA-dependent lipid-mediated protein anchoring to the cell surface, HvPssA and HvPssD. ArtA, HvPssA, and HvPssD, as well as SLG, showed midcell localization during growth and cytokinesis, indicating that haloarchaeal cells confine phospholipid processing in order to promote midcell elongation. Our findings have important implications for the biogenesis of the cell surface.

## INTRODUCTION

Microbial cell surface proteins play critical roles in many important biological processes, including bioenergetics, mediation of intercellular communication, nutrient uptake, surface adhesion, and motility. Cell surface proteins also play important roles in cell elongation and shape maintenance, but how this is achieved in archaea is not well understood (1).

The structural organization of cellular surfaces is one important readout of how cells coordinate growth, morphogenesis, and division. In both bacteria and eukaryotes, a multitude of growth modes have been characterized, with cells inserting new envelope material almost all along the cell surface (2), bipolarly (3), and unipolarly (4), and in some cases, different modes can be interchangeable (5, 6). In the case of archaea, which lack a peptidoglycan cell wall, glycosylated S-layer and other proteins are commonly the sole components of the cell envelope (7, 8), where they typically show a two-dimensional (2D) crystal-like arrangement. This poses an interesting problem for archaeal surface protein organization, and currently, there are no data about the mechanisms of archaeal cell elongation control (9).

While many proteins are anchored to the cell surface via transmembrane (TM) domain insertion into the membrane, some are anchored through covalent N-terminal attachment of a lipid moiety (8). Recently, a novel mechanism was discovered whereby proteins are anchored to the membrane through a lipid moiety covalently attached to a processed C terminus. In archaeal cells, processing and lipid modification of these C-terminal anchored proteins are mediated by enzymes known as archaeosortases, with archaeosortase A (ArtA) of the model archaeon Haloferax volcanii being the most studied example (10–13). Proteins recognized and processed by H. volcanii ArtA contain a distinct C-terminal tripartite structure consisting of a conserved PGF motif, followed by a hydrophobic domain and then a stretch of positively charged residues. Molecular biological and biochemical analyses determined that ArtA does indeed process a diverse set of proteins, including both Tat and Sec substrates, that have been shown to play roles in motility and mating (10, 12). Most notably, this includes the S-layer glycoprotein (SLG), which is the sole component of the H. volcanii cell wall.

A previous *in silico* study by Haft and coworkers noted that in Methanosarcina acetivorans C2A, Methanosarcina mazei Gö1, as well as several other methanogens, the *artA* gene is located next to the gene that encodes an archaeal homolog of bacterial phosphatidylserine synthase (PssA) (14). Based on the degree of sequence similarity and its substrate specificity, the archaeal PssA homolog belongs to the PssA subclass II, similar to PssA found in Gram-positive bacteria such as Bacillus subtilis, as opposed to the PssA of Gram-negative bacteria, such as Escherichia coli, which belongs to subclass I (15). Work in B. subtilis has elucidated most of the biochemistry involved in the reaction catalyzed by PssA, which involves the transfer of a diacylglycerol moiety from a CDP-phosphatidyl lipid to l-serine to make phosphatidylserine (16, 17). Phosphatidylserine can subsequently be decarboxylated to phosphatidylethanolamine by the enzyme phosphatidylserine decarboxylase (PssD), which has been characterized from Sinorhizobium meliloti and B. subtilis (17, 18). However, unlike bacterial PssA, *in vitro* study of the archaeal PssA homolog from Methanothermobacter thermautotrophicus (MTH_1027) revealed that this protein catalyzes the transfer of the archaetidic acid moiety of CDP-archaeol onto the hydroxyl group of l-serine to form the polar lipid archaetidylserine (CDP-2,3-di-*O*-geranylgeranyl-sn-glycerol:l-serine *O*-archaetidyltransferase) (15). Mirroring the phosphatidylethanolamine biosynthesis reaction in bacteria, it was postulated that archaetidylserine could also undergo decarboxylation to archaetidylethanolamine by an archaeal PssD homolog, a putative archaetidylserine decarboxylase.

Distant homologs to PssA and PssD are encoded in the H. volcanii genome, which we refer to as HvPssA (HVO_1143) and HvPssD (HVO_0146), respectively. In this study, we show that HvPssA and HvPssD are involved in ArtA-dependent C-terminal protein maturation, which involves proteolytic cleavage and lipid anchoring. An interplay between ArtA, HvPssA, and HvPssD is further supported by their colocalization at midcell. These analyses reveal, to the best of our knowledge for the first time, that cell elongation happens from the midcell in archaea.

## RESULTS

### Synteny of *artA*, *pssA*, and *pssD* genes in *Methanosarcina* strains.

Based on the juxtaposition of the genes encoding the archaeosortase (*artA*) and the putative membrane lipid biosynthesis archaetidylserine synthase (*pssA*) in *Methanosarcina* spp. (14), we hypothesized that homologs of the H. volcanii PssA (HvPssA) might be involved in the lipidation of ArtA substrates.

**FIG 1:**
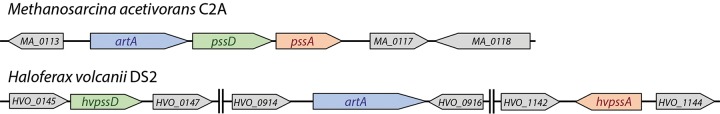
Schematic representation of *artA*, *pssA*, and *pssD* distribution across *Euryarchaeota*. Shown are *M. acetivorans* (top) and H. volcanii (bottom) genomic organization of *artA* and genes encoding homologs to PssA (HvPssA) and PssD (HvPssD). The *M. acetivorans artA*, *pssA*, and *pssD* gene lengths are 834 bp, 627 bp, and 744 bp, respectively. The H. volcanii *artA*, *hvpssA*, and *hvpssD* gene lengths are 912 bp, 672 bp, and 606 bp, respectively.

Upon further *in silico* analyses, we were intrigued that in several *Methanosarcina* species, the *artA* and *pssA* genomic region flanked a homolog of the *pssD* gene (Fig. 1; see also Fig. S1 in the supplemental material), probably encoding archaetidylserine decarboxylase, suggesting that they could be involved in consecutive steps of a proposed archaetidylethanolamine biosynthetic pathway. We decided to extend our studies of ArtA-dependent protein processing in H. volcanii toward the orthologs HvPssA (HVO_1143) and HvPssD (HVO_0146), even though the genes are not clustered with ArtA (HVO_0915) in this species (Fig. 1). The three genes are unclustered, not only in *Haloferax* spp. but also in more than 70 haloarchaeal genomes.

10.1128/mBio.00349-20.1 FIG S1 Inferred synteny between *artA, pssA*, and *pssD* homologs in *Methanomicrobiales*. Colocalization of *artA* genomic regions from 57 independent genomes using Syntax (47) showed significant synteny between *artA-pssA-pssD* homologs in 44 instances. (A) Alignment of 16 examples of *artA-pssA-pssD* clusters. (B) Alignment of 11 examples where *artA* is not clustered with *pssA* and *pssD*. Download FIG S1, TIF file, 2.3 MB. Copyright © 2020 Abdul-Halim et al. 2020 Abdul-Halim et al. This content is distributed under the terms of the Creative Commons Attribution 4.0 International license.

It should be noted that while the *Methanosarcina* spp. and H. volcanii PssA and PssD homologs share a significant similarity, these proteins have only 30 to 35% sequence identity to the *in vitro*-characterized enzymes from Methanobacterium thermautotrophicus, S. meliloti, and B. subtilis (15, 17, 18). Thus, it is possible that the H. volcanii proteins may act on variants of the substrates processed by these experimentally characterized homologs.

### H. volcanii Δ*hvpssA* and Δ*hvpssD* mutant cells exhibit growth, morphology, and motility phenotypes similar to those of the Δ*artA* mutant strain.

In order to determine whether HvPssA and/or HvPssD are involved in the archaeosortase-dependent processing pathway, we generated H. volcanii *hvpssA* and *hvpssD* deletion mutants (Fig. S2) using the pop-in/pop-out method (19).

10.1128/mBio.00349-20.2 FIG S2 H. volcanii HvPssA and HvPssD are not essential under standard laboratory growth conditions. PCR amplification using oligonucleotides against the flanking regions located approximately 700 bp upstream and 700 bp downstream of *hvpssA* or *hvpssD* (outer oligonucleotide) and oligonucleotides specific for the *hvpssA* or *hvpssD* gene (inner oligonucleotide). The template DNA was isolated from H53 wild-type (WT), Δ*hvpssA* mutant, and Δ*hvpssD* mutant strains. Download FIG S2, TIF file, 1.5 MB. Copyright © 2020 Abdul-Halim et al. 2020 Abdul-Halim et al. This content is distributed under the terms of the Creative Commons Attribution 4.0 International license.

**FIG 2:**
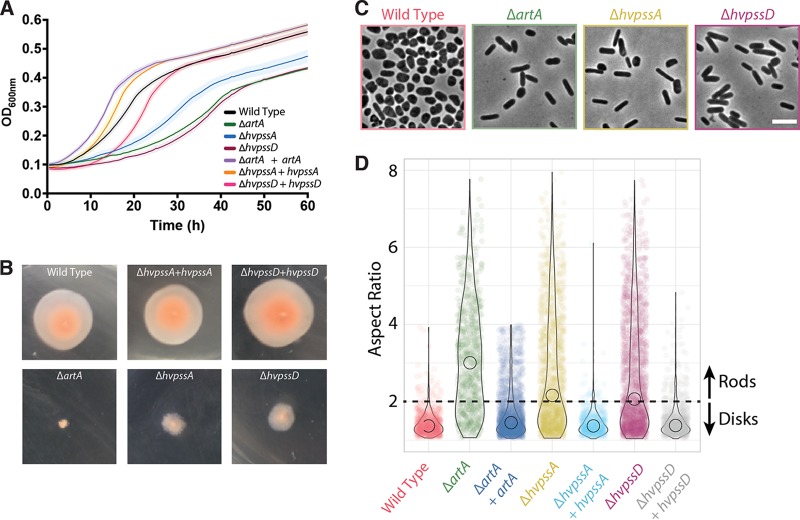
Absence of HvPssA or HvPssD leads to defects in growth, cell morphology, and motility. (A) Wild-type (strain H53) and Δ*artA*, Δ*hvpssA*, and Δ*hvpssD* mutant cells were grown with shaking in 96-well plates with a total volume of 200 μl of liquid semidefined CA medium, and the growth of six biological replicates was monitored at the OD_600_, with recordings taken every 30 min. For complementation analysis, *artA*, *hvpssA*, or *hvpssD* was expressed from pTA963 under the tryptophan-inducible p.*tna* promoter. The wild-type and deletion strains were transformed with an empty pTA963 plasmid as a control. (B) The wild-type (strain H53) and *artA*, *hvpssA*, or *hvpssD* deletion and complementation strains from individual colony on solid agar plates were individually stab inoculated with a toothpick into semisolid 0.35% agar in CA medium supplemented with tryptophan, followed by incubation at 45°C. (C) Phase-contrast images were taken from wild-type and mutant cells during mid-exponential-growth phase (OD_600_, 0.3) and immobilized under 0.5% agarose pads. (D) Violin distributions of aspect ratio measurements from single cells. Rodlike (aspect ratio, >2) cells were prevalent to disklike (aspect ratio, <2) cells in the mutant strains compared to the wild type. Biological replicates were collected on three different days, and data were analyzed from >1,000 cells under each condition by automated image segmentation. Scale bars = 5 μm.

We had previously shown that the H. volcanii Δ*artA* mutant strain exhibits various severe phenotypic defects (e.g., poor growth, atypical morphology, and impaired motility), perhaps due, at least in part, to defective processing of the SLG, an ArtA substrate (10). S-layer disruption may affect cell stability and may interfere with morphology. Also, as archaea do not have a cell wall, the S-layer may take over the function as a stator for the archaellar motor. The latter is supported by the fact that ArlF and ArlG (formerly referred to as FlaF and FlaG, respectively) were shown to interact with the S-layer glycoprotein (20, 21). We thus subjected the Δ*hvpssA* and Δ*hvpssD* mutant strains to analysis for these physiological responses. Both deletion strains exhibit a growth defect similar to that of the Δ*artA* mutant strain compared to the H53 parent strain used in these studies (Fig. 2A). For all genes, normal growth is rescued by complementation, expressing the deleted gene in *trans* from a plasmid. Moreover, the Δ*hvpssA* and Δ*hvpssD* mutant strains are partially impaired in motility; the defect, however, is less severe than that in the Δ*artA* mutant. While no halo is observed after 5 days of Δ*artA* mutant incubation at 45°C, a small halo is formed by the Δ*hvpssA* and Δ*hvpssD* mutant strains (Fig. 2B).

Light microscopic examination of the parental strain at mid-exponential-growth stage shows predominantly disk-shaped cells, while the Δ*artA* mutant cells exhibit a predominantly rod-shaped phenotype (Fig. 2C). Again, the Δ*hvpssA* and Δ*hvpssD* mutant strains show a similar but less severe phenotype. The vast majority of cells from cultures are rods, but we have observed a few disk-shaped cells in liquid cultures for each of these strains. This phenotype is fully complemented, and disk-shaped cells are observed when HvPssA and HvPssD are expressed in *trans* in the Δ*hvpssA* and Δ*hvpssD* mutant strains, respectively (Fig. S3A).

10.1128/mBio.00349-20.3 FIG S3 Cellular aspect ratio as a quantitative readout for rod versus disk morphologies in H. volcanii populations. (A) Qualitative representation of different phenotypes from wild-type and mutant cells. (B) Example of the threshold detection filter employed to quantitate the fractions of disks (green) and rods (purple) from phase-contrast images. (C) Violin plots representing the distributions of disks (aspect ratio, <2) and rods (aspect ratio, >2) in different strain populations during stationary-growth phase. (D) ArtA-, HvPssA-, and HvPssD-msfGFP cells have similar morphological profiles compared to wild-type cells. Scale bars = 5 μm. Download FIG S3, TIF file, 2.8 MB. Copyright © 2020 Abdul-Halim et al. 2020 Abdul-Halim et al. This content is distributed under the terms of the Creative Commons Attribution 4.0 International license.

To properly quantify the differences between the wild-type and mutant strains, we segmented cell images and measured the aspect ratio from each population, which is the ratio between the longer and shorter axes of the cell. Subsequently, we observed that H. volcanii rods have an aspect ratio above 2, while disks predominantly fall under this cutoff value (Fig. S3B). Using this arbitrary threshold, we automatedly determined the proportions of rods from wild-type and mutant cells (Fig. 2D and S3C), confirming our initial observation that the Δ*artA*, Δ*pssA*, and Δ*pssD* mutant populations are significantly enriched in rod-shaped cells compared to the wild type.

Thus, Δ*hvpssA* and Δ*hvpssD* mutant strains exhibit phenotypes similar to that of the Δ*artA* mutant strain for three independent physiological effects, supporting the hypothesis that the encoded proteins are involved in the ArtA-dependent processing pathway.

### HvPssA and HvPssD are required for SLG lipid modification.

**FIG 3:**
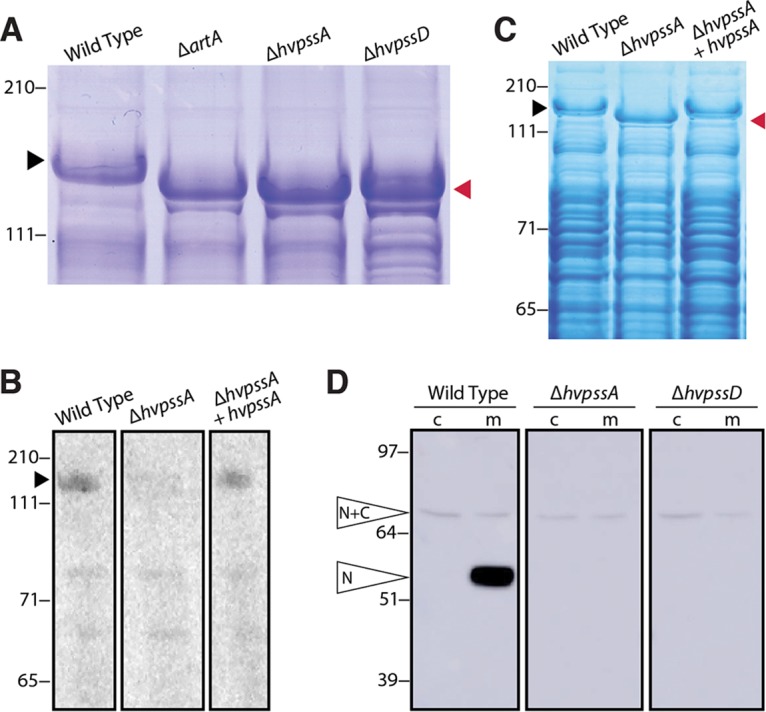
HvPssA and HvPssD are critical for HVO_0405 C-terminal processing and SLG lipidation. (A) Coomassie-stained LDS-PAGE gel of cell extracts from H. volcanii H53 (wild-type [WT]) and Δ*artA*, Δ*hvpssA*, and Δ*hvpssD* mutant strains. The Δ*artA*, Δ*hvpssA*, and Δ*hvpssD* mutant SLG (red arrowhead) exhibited a mobility shift compared to the WT SLG (black arrowhead). (B) Fluorography of protein extracts isolated from H53 (WT), Δ*hvpssA* mutant, and *hvpssA* complementation (Δ*hvpssA* + *hvpssA*) cells grown in the presence of 1 μCi/ml [^14^C]mevalonic acid. Significant labeling of SLG (black arrowhead) is only detected in the WT and *hvpssA* complementation (Δ*hvpssA* + *hvpssA*) extracts. (C) Coomassie staining of the gel used for fluorography. The SLG mobility shift in the Δ*hvpssA* mutant (red arrowhead) is reverted upon *hvpssA* expression in *trans*. (D) Western blot analysis of cytoplasmic (c) and membrane (m) fractions of H53 (WT), Δ*hvpssA* mutant, and Δ*hvpssD* mutant strains expressing, in *trans*, HVO_0405-6×His. The N-terminal domain of HVO_0405 was detected using anti-HVO_0405-N-term antibodies. Hvo_0405 not processed by ArtA and the N-terminal HVO_0405 processed by ArtA are labeled “N+C” and “N,” respectively. The C-terminal domain, which carries a His tag, has not been analyzed in this experiment. Numbers indicate molecular mass in kilodaltons.

To determine whether the drastic cell morphology transitions and the phenotypic similarities between the Δ*hvpssA*, Δ*hvpssD*, and Δ*artA* mutants are due to the inhibition of the covalent lipid modification of ArtA substrates, we investigated the lipidation of the SLG mediated by ArtA (11). Initially, as an indirect analysis, we examined the effect of *hvpssA* and *hvpssD* deletions on SLG electrophoretic mobility in a lithium dodecyl sulfate (LDS)-PAGE gel, as a mobility shift is observed in an *artA* deletion strain (11). While the similarities of Coomassie-stained band intensities for SLG isolated from the Δ*artA*, Δ*hvpssA*, and Δ*hvpssD* mutant strains and their parent strains indicate a similar SLG abundance, electrophoretic mobility demonstrates similar migration shifts of the SLG isolated from all three deletion strains compared to the SLG from the parent strain (Fig. 3A). These shifts are reverted by complementation with the respective gene in *trans* (Fig. S4A). To corroborate this observation, we set out to directly measure the lipid modification of SLG in the Δ*hvpssA* mutant strain. These experiments proved that lipid labeling of the SLG with radiolabeled mevalonic acid, an archaeal lipid precursor, is severely impaired in the Δ*hvpssA* mutant strain compared to the parent strain (Fig. 3B) and that this phenotype can be complemented by expressing HvPssA in *trans*. This is conclusive evidence for HvPssA being closely or even directly coupled with ArtA-dependent protein lipidation. Because the phenotypes of the Δ*hvpssD* mutant strain closely match those of the Δ*hvpssA* mutant strain in all other assays, including the indirect gel-based assay for SLG modification (Fig. 3A to C), it is highly likely that HvPssD is also involved in ArtA-dependent SLG lipidation.

10.1128/mBio.00349-20.4 FIG S4 Lack of lipidation results in SLG mobility shift and prevents ArtA-dependent HVO_0405 processing. (A) Coomassie-stained LDS-PAGE gel of cell extracts from H. volcanii H53 (WT), Δ*artA* mutant, Δ*hvpssA* mutant, Δ*hvpssD* mutant, and complementation strains. The observed mobility shift of SLG bands in the deletion mutants (green arrowhead) was reverted in the complementation strains by ectopic expression (black arrowhead). (B) Western blot analysis of membrane (m) and supernatant (s) fractions of the H53 (WT) and Δ*artA*, Δ*hvpssA*, and Δ*hvpssD* mutant strains expressing HVO_0405-6×His in *trans*. The N-terminal domain of HVO_0405 was detected using anti-HVO_0405-N-term antibodies. The processed N-terminal HVO_0405, marked as “N,” can only be detected in the membrane fraction of the WT. The bands in the supernatant fraction of all mutant strains are not related to ArtA-dependent processing (i.e., cleaved but not lipidated HVO_0405), as they are detected for the Δ*artA* mutant as well. Instead, they probably indicate proteolytic degradation products of HVO_0405. Numbers indicate molecular mass in kilodaltons. Download FIG S4, TIF file, 2.3 MB. Copyright © 2020 Abdul-Halim et al. 2020 Abdul-Halim et al. This content is distributed under the terms of the Creative Commons Attribution 4.0 International license.

### HvPssA and HvPssD are required for proteolytic ArtA-substrate processing.

To determine whether HvPssA/HvPssD-dependent lipidation is required for ArtA-dependent C-terminal processing, we investigated the C-terminal proteolytic cleavage of a second ArtA substrate. As a reporter, we used the strain-specific domain fusion protein HVO_0405, with its centrally located cleavage site, because the cleaved and uncleaved versions of HVO_0405 can be easily distinguished by LDS-PAGE separation and subsequent immunostaining (12).

Cleavage of C-terminally His-tagged HVO_0405, expressed in *trans*, does not occur in the Δ*hvpssA* and Δ*hvpssD* mutant strains. In contrast, this ArtA substrate is cleaved in the corresponding parent strain, as is evident from our Western blot analysis of membrane fractions using antibodies against the N-terminal part of HVO_0405 (Fig. 3D and S4B). Blocking proteolytic cleavage does not lead to an increased amount of the full-length form. The same has been observed in an Δ*artA* mutant (12) and has been attributed to the instability of the domain-fused version of this protein.

### Midcell localization of ArtA, HvPssA, and HvPssD promotes cell site-specific lipidation *in vivo*.

**FIG 4:**
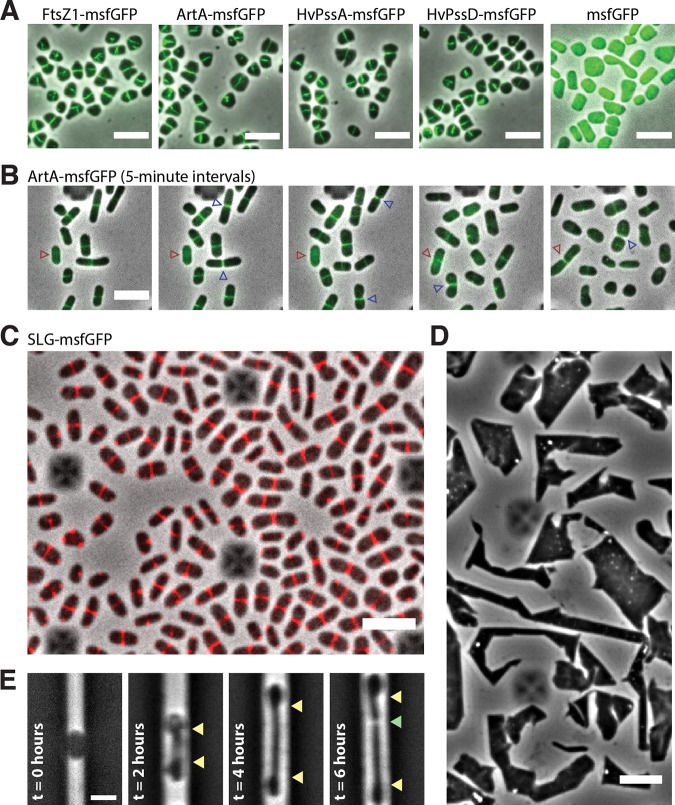
Midcell localization of the lipid-anchoring and processing machinery in H. volcanii. (A) Snapshots of merged phase-contrast (gray) and fluorescein isothiocyanate (FITC; green) channels of cells expressing FtsZ1-msfGFP, ArtA-msfGFP, HvPssA-msfGFP, HvPssD-msfGFP, and soluble msfGFP. Cells were immobilized under 0.5% agarose pads prepared with CA medium. (B) Time-lapse images of cells growing inside a CellASIC microfluidic device. Images of merged phase-contrast (gray) and FITC (green) channels were taken every 5 min for 12 h. Blue arrowheads indicate cell division events, while red arrowheads label one example of a cell elongating only after the arrival of ArtA-msfGFP to the midcell. (C) Snapshot of SLG-msfGFP (red) midcell localization. (D) Phase-contrast images of H. volcanii cells under prolonged overexpression (24 h) of the SLG-msfGFP fusion. (E) H. volcanii cells reshape and elongate preferentially at the midcell during protoplast recovery. Cells were loaded into the microfluidic chamber, and the S-layer was chemically removed by the addition of 1 mg/ml proteinase K and recovered with fresh medium (*t* = 0 h). Yellow arrowheads indicate the cell area extended until cell division (green arrowhead). Scale bars = 5 μm.

Given the dependence of ArtA activity on HvPssA and HvPssD and their importance to cell growth and morphogenesis, we decided to investigate whether ArtA, HvPssA and HvPssD were recruited to perform at specific regions of H. volcanii cells. Strikingly, ArtA, HvPssA, and HvPssD tagged with monomeric superfolder green fluorescent protein (msfGFP) localize at midcell (Fig. 4A). As controls, we also imaged a tagged version of FtsZ1, that has previously been shown to localize to midcell and was speculated to participate in cell division (22), as well as msfGFP not fused to any protein. FtsZ1-msfGFP shows a localization pattern almost identical to those of ArtA, HvPssA, and HvPssD, while the msfGFP protein by itself is not recruited to midcell (Fig. 4A). Furthermore, we confirmed that cells carrying msfGFP-tagged versions of ArtA, HvPssA, and HvPssD present morphological profiles similar to that of the wild type and thus must be functional (Fig. S3D). Finally, time-lapse videos of cells cultivated within microfluidics suggest that ArtA, HvPssA, and HvPssD proteins are recruited to the midcell right after daughter cells are born and persist for most of the cell cycle, including during cytokinesis (Fig. 4B, blue arrowhead, and Movie S1).

10.1128/mBio.00349-20.6 MOVIE S1 FtsZ1, ArtA, HvPssA, and HvPssD midcell localization and dynamics throughout the cell cycle in H. volcanii. Time-lapse images were acquired with cells growing inside a CellASIC microfluidic device. Images of merged phase-contrast (gray) and FITC (green) channels were taken every 5 minutes for 12 h. Scale bar = 5 μm. Download Movie S1, MOV file, 15.0 MB. Copyright © 2020 Abdul-Halim et al. 2020 Abdul-Halim et al. This content is distributed under the terms of the Creative Commons Attribution 4.0 International license.

Considering that deletions of *artA*, *hvpssA*, and *hvpssD* each drastically perturbed growth (Fig. 2A), induced cells to stay in a rodlike shape (Fig. 2C), and do not seem to play an essential role in cell division, we hypothesize that the lipid anchoring of ArtA substrates specifically at midcell might be important for cell elongation and morphogenesis. Interestingly, we also noticed a correlation between the presence of ArtA, HvPssA, and HvPssD at the midcell and actual cell elongation in the cell population (Fig. 4B, red arrowhead). To investigate this further, we expressed a second copy of the SLG which was tagged with msfGFP, a GFP variant shown to be fluorescent upon Sec-dependent transport in bacteria (23). Interestingly, the SLG-msfGFP fusion accumulated at the midcell site instead of localizing around the cell envelope (Fig. 4C and Movie S2). Thus, even though both versions of SLG, tagged and untagged, are synthesized, they do not seem to coassemble across the S-layer. Additionally, overexpression of the SLG-msfGFP fusion caused severe growth and morphological defects (Fig. 4D). Interestingly, cells rapidly recovered in less than 1 day (approximately 6 generations) following depletion of the SLG-msfGFP, returning to their normal cell shape (Fig. S5). It might be possible that secreted SLG-msfGFP cannot be incorporated into the 2D proteinaceous crystal array. This scenario could explain the observed SLG-msfGFP foci at the poles (Fig. 4C), as an excess of secreted but unassembled SLG protein could aggregate at the pole, where the S-layer array must show discontinuities. However, independent of whether this SLG construct was secreted or not, our results strongly suggest that nascent SLG is targeted specifically to the midcell.

10.1128/mBio.00349-20.5 FIG S5 Prolonged overexpression of the SLG-msfGFP fusion causes severe growth and morphological defects but does not impair H. volcanii viability. aBL118 cells carrying the pABHV10 plasmid (pTA962::csg-msfGFP) have normal morphology prior to the induction of SLG-msfGFP (left). Upon the addition of 2.5 mM of tryptophan in liquid culture, cells rapidly lose shape (center) but are still alive and able to recover following depletion of SLG-msfGFP fusion (right). Scale bars = 10 μm. Download FIG S5, TIF file, 2.0 MB. Copyright © 2020 Abdul-Halim et al. 2020 Abdul-Halim et al. This content is distributed under the terms of the Creative Commons Attribution 4.0 International license.

10.1128/mBio.00349-20.7 MOVIE S2 SLG-msfGFP midcell localization in H. volcanii cells. Time-lapse images were acquired with cells growing inside a CellASIC microfluidic device. Images of merged phase-contrast (gray) and FITC (red) channels were taken every 5 minutes for 12 h. During the first 6 h, cells were grown and imaged in the absence of tryptophan (no induction). Between 6 and 12 h, tryptophan was added to a final concentration of 500 μM, inducing the SLG-msfGFP expression (induction). Scale bar = 5 μm. Download Movie S2, MOV file, 2.7 MB. Copyright © 2020 Abdul-Halim et al. 2020 Abdul-Halim et al. This content is distributed under the terms of the Creative Commons Attribution 4.0 International license.

Last, we investigated the morphological transitions in H. volcanii protoplast cells during *de novo* S-layer synthesis. If the midcell-confined ArtA, HvPssA, and HvPssD are in fact promoting the lipidation of recently secreted SLG molecules at the cell surface, one would be able to observe midcell-localized reshaping during protoplast recovery. As expected, protoplasts generated by the addition of proteinase K either within microfluidics or in bulk cultures adopt a round-like shape (Fig. 4E, left), suggesting that the S-layer might be the structure that ultimately determines the archaeal cell shape. As the protease is washed out with fresh medium, cells rapidly reshape exclusively from the midcell position (Fig. 4E). Altogether, our observations suggest that the midcell area in H. volcanii cells is not only dedicated to cell division but is also a central hub for outbound cell extension and other cellular processes.

## DISCUSSION

Our data confirmed the hypothesized involvement of the lipid biosynthesis enzyme homologs HvPssA and HvPssD in the C-terminal posttranslational modifications of ArtA substrates. With respect to physiological effects, the Δ*hvpssA* and Δ*hvpssD* mutants showed similar but slightly less severe phenotypes than that resulting from the deletion of *artA* (Fig. 2). We furthermore experimentally determined the effects of *hvpssA* and *hvpssD* deletions on proteolytic cleavage and lipid labeling (Fig. 3).

Since the gene encoding HVO_0405 resulted from the fusion of two previously independent genes, this protein provided us with an excellent tool for the analysis of ArtA-related proteolysis in H. volcanii, as the size difference between precursor- and ArtA-processed mature proteins is large enough to be detected using immunostaining (12). This allowed us to clearly demonstrate that ArtA-dependent proteolytic cleavage is blocked when either *hvpssA* or *hvpssD* is deleted in H. volcanii. This block could be bypassed by plasmid-based gene complementation. These results strongly suggest that lipidation and proteolysis are intricately connected with proteolytic cleavage only occurring if the modifying lipid and/or HvPssA or HvPssD is present. By sequence homology, H. volcanii HvPssA is predicted to be involved in lipid biosynthesis, specifically, the generation of the polar lipid archaetidylserine from CDP-archaeol. While the functionally characterized homolog (from *M. thermautotrophicus*) is only distantly related (30% to 35% sequence identity), the confirmed involvement of HvPssD strongly supports the hypothesis that HvPssA and HvPssD generate archaetidylethanolamine.

**FIG 5:**
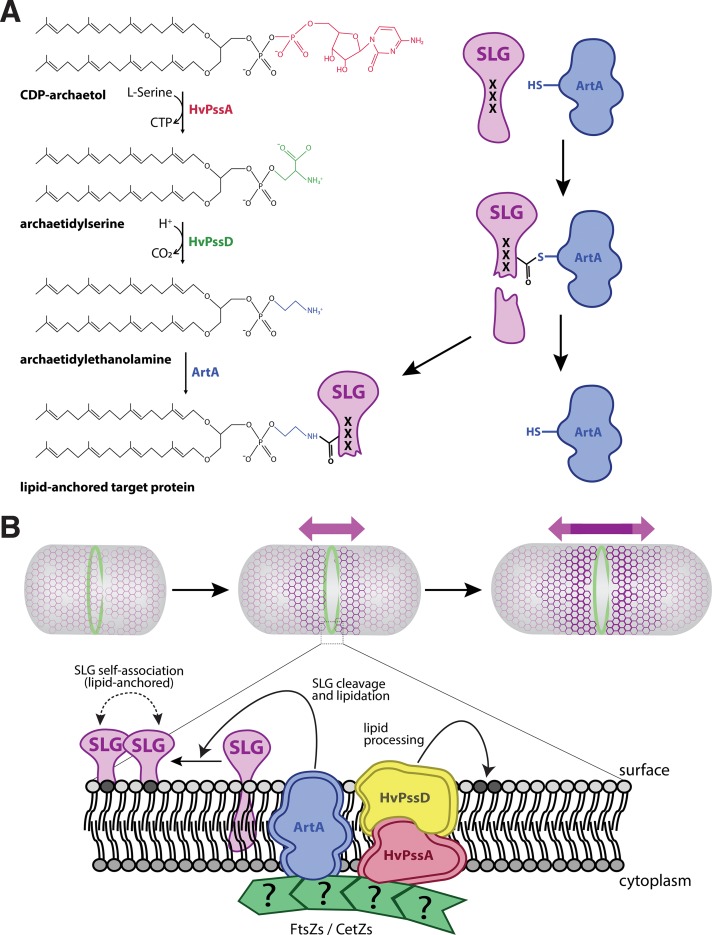
A model for lipid attachment and cell growth involving HvPssA, HvPssD, and ArtA. (A) In our speculative model, CDP-archaeol is converted to archaetidylethanolamine in two steps involving HvPssA and HvPssD. ArtA acts as a peptidase and covalently links its active-site cysteine to a newly generated C terminus of its target protein, simultaneously releasing the C-terminal peptide. Then, the free amino group of ethanolamine attacks the thiocarboxylate, which marks the covalent attachment of the target protein to the ArtA active-site cysteine. This results in covalent attachment of the lipid to the C terminus of the target protein as a carboxamide, simultaneously releasing ArtA. The process of cleavage and lipidation is dependent on HvPssA and HvPssD, either by binding of archaetidylethanolamine to ArtA or by protein-protein interaction between ArtA and HvPssA or HvPssD. (B) Recruitment of ArtA, HvPssA, or HvPssD to the midcell promotes anchoring of surface proteins and insertion of new SLG into the S-layer at midcell, contributing to cell elongation and division.

Interestingly, a recent characterization of a bacterial rhombosortase, a nonhomologous analog of archaeosortase (24), showed direct involvement of a glycerophosphoethanolamine-containing moiety in the process. Analogously, lipid-attached ethanolamine may be directly involved in the membrane anchoring of ArtA substrates. In this scenario, instead of contributing directly to the ArtA-mediated substrate cleavage and/or lipid anchoring, HvPssA and HvPssD catalyze the final steps in a pathway that generates archaetidylethanolamine, a substrate required by this process. This opens the way for another hypothesis regarding the ArtA reaction mechanism. In this scenario, ArtA acts similarly to sortase A in bacteria, wherein ArtA cleaves the substrate through thioesterification, forming a thioester acyl‐enzyme intermediate, which is consistent with the identification of Cys-173 as an active-site residue (13). The nucleophilic attack of an amine resolves this intermediate, but instead of a pentaglycine branched lipid II, the reactive amine nucleophile is archaetidylethanolamine (Fig. 5A). Such a mechanism would directly result in a covalently modified protein C terminus. While an archaetidylethanolamine lipid was reported to be absent from Halobacterium salinarum or Haloarcula marismortui (25), it is present in H. volcanii and several other haloarchaea, albeit at various abundances (26).

As lipid analysis does not cope with protein-bound lipids, the low concentration of archaetidylethanolamine is not surprising, even though the SLG is highly abundant and archaetidylethanolamine may be used as its membrane anchor. Nevertheless, a detectable amount of archaetidylethanolamine in the H. volcanii membrane suggests the functional roles of HvPssA and HvPssD, catalyzing the synthesis of archaetidylserine and its decarboxylation to archaetidylethanolamine, respectively. These enzymes perhaps associate with or even form a complex with ArtA, resulting in a majority of the synthesized archaetidylethanolamines to be immediately used to modify the SLG and other ArtA substrates for their membrane anchoring upon C-terminal processing. Thus, only a small amount may be left free in the membrane. The lipid attached to an EDTA-soluble fraction of the SLG has been analyzed by mass spectrometry and was identified as archaetidic acid (27). However, as the lipid has been released from the protein by alkaline hydrolysis, this procedure may have hydrolyzed and thus removed the ethanolamine headgroup.

While investigating the interdependence between ArtA and HvPssA or HvPssD, we observed the recruitment of these proteins to the midcell in H. volcanii (Fig. 4A). Considering these data and the observed midcell localization of newly inserted SLG molecules (Fig. 4C), we propose a model for S-layer assembly, lipidation, and growth in haloarchaea (Fig. 5B). First, SLG is recruited to the midcell, where it is transported across the cytoplasmic membrane in a Sec-dependent manner (28). Following secretion, SLG is processed and linked to archaetidylethanolamine by ArtA, requiring HvPssA or HvPssD for archaetidylethanolamine synthesis and/or interaction with ArtA.

There are still key aspects of haloarchaeal growth and shape control that are not addressed by our model. For example, it is still not clear how the deletion of either *artA*, *hvpssA*, or *hvpssD* generates a rod-shaped cell population (Fig. 2C). Furthermore, although it has been shown that the S-layer is not essential in other archaea (29, 30), ectopic overexpression of our SLG-msfGFP fusion drastically impacted the morphology of H. volcanii cells (Fig. 4D) beyond the lack of SLG processing and lipidation (11). Therefore, it is possible that our SLG-msfGFP fusion is actually blocking the transport of or interaction with other yet-unknown surface proteins essential for shape maintenance. The concept of having different classes of surface-modifying proteins counteracting each other has been demonstrated in bacteria, where different classes of penicillin binding proteins (PBPs) act on the peptidoglycan cell wall to control cell width homeostasis in rod-shaped cells (31).

Interestingly, just like ovococcoid bacteria that are capable of midcell elongation and lack a clear dedicated elongation machinery, haloarchaea may be also employing cytoskeletal polymers to direct different subcomplexes for cell elongation and cell division (32). This evidence is even more striking for coccoid bacteria, for which a single point mutation in FtsZ is able to induce cell elongation in Staphylococcus aureus (33). However, haloarchaea might have conserved at least two distinct elongation modes in addition to cell division, generating disklike and rodlike populations (34). This scenario would also corroborate the morphological malleability of haloarchaea, being capable of assuming unusual shapes like triangles and squares (35, 36).

In spite of the bacteria that use the localization of specialized proteins to promote midcell elongation, it is important to point out that these mechanisms are likely not evolutionarily related to the proposed haloarchaeal S-layer lipidation and cell elongation. First, even though there are examples of bacterial species with an S-layer that carries out peptidoglycan cell wall synthesis at the midcell (6), their new S-layer material is inserted as patches distributed all around the cell (37, 38). Second, the lack of conservation in the protein architecture between archaeal and bacterial S-layers argues that they may have emerged independently of each other (8, 39).

In conclusion, by applying a set of different experimental approaches, we have confirmed that two putative lipid biosynthesis enzymes, HvPssA and HvPssD, are involved in the proteolytic cleavage and lipid labeling of ArtA substrates specifically at midcell. We have also proposed, to the best of our knowledge, the first molecular model for archaeal cell elongation.

## MATERIALS AND METHODS

### Strains and growth conditions.

The plasmids and strains used in this study are listed in Table 1. H. volcanii strain H53 and its derivatives were grown at 45°C in semidefined Casamino Acids (CA) medium supplemented with tryptophan (50 μg ml^−1^ final concentration) (40). Cells were cultivated either in liquid medium (orbital shaker at 250 rpm) or on solid 1.5% agar. Difco agar and Bacto yeast extract were purchased from Becton, Dickinson, and Company. Peptone was purchased from Oxoid. To ensure equal agar concentrations in all plates, agar was completely dissolved in the media prior to autoclaving, and the autoclaved media were stirred before plates were poured. Escherichia coli strains were grown at 37°C in NZCYM medium (Fisher Scientific) supplemented with ampicillin (100 μg/ml).

**TABLE 1:** Strains and plasmids used in this work

Plasmid or strain	Genotype and/or description^*a*^	Reference or source
Plasmids		
pTA131	pBluescript II with BamHI-XbaI fragments from pGB70 harboring *p.fdx-pyrE2*, Amp^r^	19
pTA963	*pyrE2* and *hdrB* markers, Trp-inducible (*p.tna*) promoter, Amp^r^	43
pFH25	pTA963 carrying *hvo_0405* with C-terminal 6×His tag	12
pFH38	pTA131 carrying 700 bp upstream and 700 bp downstream *hvpssA* flanking region	This work
pFH43	pTA131 carrying 700 bp upstream and 700 bp downstream *hvpssD* flanking region	This work
pFH39	pTA963 carrying *hvpssA* with C-terminal 6×His tag	This work
pFH44	pTA963 carrying *hvpssD* with C-terminal 6×His tag	This work
pFH55	pTA963 carrying *hvo_0405* N-terminal LVIVD domain with C-terminal 6×His tag	This work
Strains		
DH5α	E. coli F^−^ ϕ80Δ*lacZ*ΔM15 (*lacZYA*-*argF*)*U169 recA1 endA1 hsdR17*(*r*_K_^−^ *m*_K_^−^) *phoA supE44 thi-1 gyrA96 relA1*	Invitrogen
DL739	E. coli MC4100 *recA dam-13*::Tn*9*	48
H26	H. volcanii Δ*pyrE2*	19
H53	H. volcanii Δ*pyrE2* Δ*trpA*	19
FH27	H53/pTA963::*hvo_0405*-6×His	12
FH28	H53 Δ*artA*/pTA963::*hvo_0405*-6×His	12
FH55	H53 Δ*hvpssA*/pTA963	This work
FH56	H53 Δ*hvpssA*/pTA963::*hvpssA-*6×His	This work
FH57	H53 Δ*hvpssA*/pTA963::*hvo_0405-*6×His	This work
FH69	H53 Δ*hvpssD*/pTA963	This work
FH70	H53 Δ*hvpssD*/pTA963::*hvpssD-*6×His	This work
FH71	H53 Δ*hvpssD*/pTA963::*hvo_0405-*6×His	This work
FH77	H53 Δ*hvo_0405*/pTA963::N-term-*hvo_0405*-6×His	This work
aBL128	Δ*pyrE2 artA*::*artA-msfGFP-pyrE2*	This work
aBL129	Δ*pyrE2 hvpssD*::*hvpssD-msfGFP-pyrE2*	This work
aBL183	Δ*pyrE2 hvpssA*::*hvpssA-msfGFP-pyrE2*	This work
aBL131	Δ*pyrE2 ftsZ1*::*ftsZ1-msfGFP-pyrE2*	This work
aBL184	Δ*pyrE2*/pTA962::msfGFP(SW)	This work
aBL118	Δ*pyrE2*/pTA962::*csg*-*msfGFP*(SW)	This work

a Amp^r^, ampicillin resistant.

### Plasmid preparation and H. volcanii transformation.

DNA polymerase, DNA ligase, and restriction enzymes were purchased from New England BioLabs. Plasmids were initially transformed into E. coli DH5α cells. Plasmid preparations were performed using the QIAprep Spin miniprep (Qiagen) kit. Prior to H. volcanii transformation, plasmids were transformed into the Dam^−^ E. coli strain DL739. H. volcanii transformations were performed using the polyethylene glycol (PEG) method (40). All oligonucleotides used to construct the recombinant plasmids are listed in Table S1.

10.1128/mBio.00349-20.8 TABLE S1 Oligonucleotides used in this work. Download Table S1, DOCX file, 0.1 MB. Copyright © 2020 Abdul-Halim et al. 2020 Abdul-Halim et al. This content is distributed under the terms of the Creative Commons Attribution 4.0 International license.

### Generation of chromosomal *hvpssA* and *hvpssD* deletions in H53.

Chromosomal deletions were generated by homologous recombination (pop-in/pop-out), as previously described (19). Plasmid constructs for use in the pop-in/pop-out knockout process were generated by using overlap PCR, as described previously (41), as follows: approximately 700 nucleotides flanking the *hvpssA* gene were PCR amplified and cloned into the haloarchaeal suicide vector pTA131. The *hvpssA* upstream flanking region was amplified with oligonucleotides FW_pssA_KO_XbaI and RV_pssA_up, while the *hvpssA* downstream flanking region was amplified using FW_pssA_dw and RV_pssA_KO_XhoI (oligonucleotides are listed in Table S1). The *hvpssA* upstream and downstream flanking DNA fragments were fused by PCR using oligonucleotides FW_pssA_KO_XbaI and RV_pssA_KO_XhoI, followed by cloning into pTA131 digested with XbaI and XhoI. The insertion of the correct DNA fragment into the cloning site of the recombinant plasmid was verified by sequencing using the same oligonucleotides. The final plasmid construct, pFH38, contained upstream and downstream *hvpssA* flanking regions and was transformed into the parental H53 H. volcanii strain. To confirm the chromosomal replacement event at the proper location on the chromosome, colonies derived from these techniques were screened by PCR using the FW_pssA_KO_XbaI and RV_pssA_KO_XhoI oligonucleotides. The *hvpssA* deletion mutant generated in strain H53 was designated FH38 (Table 1). For the generation of the plasmid construct for chromosomal *hvpssD* deletion, approximately 700 nucleotides flanking the *hvpssD* gene were PCR amplified and cloned into the haloarchaeal suicide vector pTA131. The upstream flanking region was amplified with oligonucleotides FW_pssD_KO_XbaI and RV_pssD_up, while the downstream flanking region was amplified using FW_pssD_dw and RV_pssD_KO_XhoI. The flanking DNA fragments were fused by PCR using oligonucleotides FW_pssD_KO_XbaI and RV_pssD_KO_XhoI, followed by cloning into pTA131 digested with XbaI and XhoI. The insertion of the correct DNA fragment into the cloning site of the recombinant plasmid was verified by sequencing using the same oligonucleotides. The final plasmid construct, pFH43, contained upstream and downstream *hvpssD* flanking regions and was transformed into the parental H. volcanii strain H53. Confirmation of *hvpssD* deletion on the chromosome was screened by PCR using the FW_pssD_KO_XbaI and RV_pssD_KO_XhoI oligonucleotides. The *hvpssD* deletion mutant generated in strain H53 was designated FH63 (Table 1).

### Construction of expression plasmids for HvPssA and HvPssD.

To construct a tryptophan-inducible H. volcanii *hvpssA* gene with C-terminal His tag, its coding region was amplified by PCR using the oligonucleotides FW_pssA_OE_NdeI and RV_pssA_OE_EcoRI_His (Table S1). Meanwhile, for the construction of the H. volcanii *hvpssD* gene with a C-terminal His tag, its coding region was amplified by PCR using the oligonucleotides FW_pssD_OE_NdeI and RV_pssD_OE_EcoRI_His (Table S1). The PCR product was cloned into the expression vector pTA963 that had been digested with NdeI and EcoRI. This places the *hvpssA* or *hvpssD* gene under the control of the inducible tryptophanase promoter (p.*tna*). The recombinant pTA963 carrying the *hvpssA* gene was designated pFH39, and the pTA963 carrying *hvpssD* was designated pFH44. To complement the Δ*hvpssA* mutant strain FH38, this strain was transformed with plasmid pFH39 to result in FH56. For complementation of the Δ*hvpssD* mutant strain FH63, this strain was transformed with plasmid pFH44 to result in FH70. The H53, Δ*hvpssA* mutant, and Δ*hvpssD* mutant strains were also transformed with the empty expression vector pTA963, which was used as a control.

### Construction of expression plasmid for SLG-msfGFP and msfGFP.

To construct a tryptophan-inducible H. volcanii *csg* gene tagged with msfGFP, a sandwich fusion was created by intercalating an msfGFP gene product amplified from a synthetic fragment (oligonucleotides oHV81 and oHV82) between the first 102 bp (the secretion signal sequence of SLG, oligonucleotides oAB500 and oAB501) and the rest of the *csg* coding region (oligonucleotides oHV83 and oAB502). The 3 fragments were then assembled by Gibson Assembly (42) together with the pTA962 plasmid (43), digested with NheI and BamHI, and transformed into DH5α cells. The same process was followed for the cloning of msfGFP alone (oligonucleotides oHV101 and oHM68). The clones were subjected to validation by PCR and Sanger sequencing (Table S1).

### Generation of chromosomal msfGFP fusions in H26.

C-terminal translational fusion constructs, including a pyrE2 cassette for selection, were generated by direct transformation of PCR fragment ensembles generated by Gibson Assembly (42), transformed directly in H. volcanii H26 cells, and selected by growth in the absence of uracil. PCR fragments from *ftsZ1* (oligonucleotides oHV3 and oHV4 for the upstream region and oHV8 and oHV9 for the downstream region), *artA* (oligonucleotides oHM91 and oHM92 for the upstream region and oHM93 and oHM94 for the downstream region), *hvpssA* (oligonucleotides oHV156 and oHV157 for the upstream region and oHV158 and oHV159 for the downstream region), and *hvpssD* (oligonucleotides oHV126 and oHV127 for the upstream region and oHV128 and oHV129 for the downstream region) were assembled to msfGFP (oligonucleotides oHM34 and oHM6) and the *pyrE2* cassette (oligonucleotides oHV6 and oHV7). Chromosomal replacements were confirmed by PCR and Sanger sequencing (Table S1).

### Immunoblotting.

Liquid cultures were grown until mid-log phase (optical density at 600 nm [OD_600_], 0.2 to 0.5), and the cells were harvested by centrifugation at 3,800 × *g* for 5 min at room temperature. Cell pellets were resuspended and lysed in 1% (vol/vol) NuPAGE lithium dodecyl sulfate (LDS) sample buffer supplemented with 100 mM dithiothreitol (DTT) and stored at –20°C. Samples were electrophoresed on 4 to 12% Bis-Tris polyacrylamide gels (Invitrogen) with NuPAGE 3-(*N*-morpholino)propanesulfonic acid (MOPS)-SDS running buffer (Invitrogen). Proteins were then transferred to polyvinylidene difluoride (PVDF) membranes (Millipore) using a semidry transfer apparatus at 15 V for 30 min (Bio-Rad). Subsequently, the membrane was washed twice in phosphate-buffered saline (PBS), blocked for 1 h in 3% bovine serum albumin (BSA) in PBS, and washed twice in PBS with 1% Tween 20 and once with PBS. For detection of the poly-His tag, the mouse anti-penta-His antibody (catalog no. 34660; Qiagen) was used at a 1:2,000 dilution in 3% BSA in PBS with sodium azide. For the secondary antibody, horseradish peroxidase (HRP)-conjugated Amersham ECL anti-mouse IgG from sheep (GE) was used at a 1:20,000 dilution in 10% nonfat milk in PBS. For the detection of HVO_0405_Nterm, the rabbit anti-HVO_0405-N-term serum (12) was used as the primary antibody at a 1:10,000 dilution in 3% BSA in PBS with sodium azide. For the secondary antibody, HRP-conjugated Amersham ECL anti-rabbit IgG from donkey (GE) was used at a 1:60,000 dilution in 10% nonfat milk in PBS.

### Lipid radiolabeling.

The H53 parent strain carrying the vector control pTA963 and the Δ*hvpssA* mutant strain carrying either the *hvpssA* expression plasmid pFH39 or the vector control pTA963 were grown in 5 ml liquid CA medium. Upon reaching mid-log phase (OD_600_, ∼0.5), 20 μl of each culture was transferred into 1 ml of fresh liquid CA medium supplemented with [^14^C]mevalonic acid (resuspended in ethanol) at a final concentration of 1 μCi/ml. Haloferax volcanii cultures were harvested after reaching mid-log phase, and proteins were precipitated from 1-ml cultures with 10% trichloroacetic acid (TCA), followed by a delipidation step to remove noncovalently linked lipid, as described previously (44, 45). The delipidated proteins were separated by 7% Tris-acetate (TA) LDS-PAGE gels. For analysis of the samples, the gel was dried onto blotting paper using a gel dryer (model 583; Bio-Rad), exposed to a phosphor screen (Molecular Dynamics) for 3 weeks, and analyzed using a Typhoon imager (Amersham Biosciences).

### Motility assays.

The motility assays of the H. volcanii H53 (parent) and the Δ*artA*, Δ*hvpssA*, and Δ*hvpssD* mutant strains carrying the plasmid expressing the complementary gene (or pTA963 as control) were performed on 0.35% agar in CA medium supplemented with tryptophan, as described previously (41). A toothpick was used to stab inoculate the agar, followed by incubation at 45°C. Halo sizes around the stab-inoculation site were measured after 3 to 5 days of incubation.

### Growth curves.

Growth curves were measured using a BioTek PowerWaveX2 microplate spectrophotometer. H. volcanii H53 (parent) and Δ*artA*, Δ*hvpssA*, and Δ*hvpssD* mutant strains carrying the plasmid expressing the complementary gene (or pTA963 as control) were first incubated in 5-ml liquid cultures in CA medium supplemented with tryptophan with continuous shaking at 45°C until suitable OD_600_ values (0.2 to 0.5) were reached. Approximately 6 μl of each culture (adjusted to correct for OD_600_ differences) was then transferred into 194 μl of fresh CA medium supplemented with tryptophan (50 μg ml^−1^ final concentration) and grown to stationary phase, with OD_600_ recordings taken every 30 min.

### Light microscopy.

The H. volcanii strains H53 (parent) and Δ*artA*, Δ*hvpssA*, and Δ*hvpssD* mutant strains carrying the plasmid expressing the complementary gene (or pTA963 as control) were inoculated from colony to 5 ml CA liquid medium and grown until they reached mid-log phase (OD_600_, ∼0.4 to 0.5). Serial liquid-to-liquid subinoculations were carried out by transferring 10 μl of the liquid culture to 5 ml fresh liquid CA medium with up to two transfers. Subsequently, 1 ml of each culture was concentrated by centrifugation at 4,911 × *g* for 1 min, and the pellets were resuspended in 10 μl of liquid CA medium. Then, 10 μl of the concentrated cells was transferred to under a 0.5% agarose pad with CA medium and observed using a Nikon Eclipse TiE inverted total internal reflection fluorescence (TIRF) microscope. ArtA-msfGFP time-lapse images were acquired by culturing H. volcanii cells inside Millipore ONIX CellASIC microfluidic plates, as previously described (35). Images were taken under 5-min intervals for 12 h in both phase-contrast and 488-nm laser channels. H. volcanii protoplasts were generated within microfluidic channels by the addition of 1 mg/ml of proteinase K (Invitrogen) in YPC medium until cells lost shape. Subsequently, the cells were washed with fresh YPC, and time-lapse images were recorded with 10-min intervals for 12 h.

### Image analysis.

All image processing, unless otherwise specified, was performed in Fiji. Automated segmentation of cells and subsequent aspect ratio measurements were performed using the MicrobeJ plugin (46). Finally, statistics and plots were generated using ggplot2 in RStudio.

### Gene synteny analysis.

We used the SyntTax server (https://archaea.i2bc.paris-saclay.fr/synttax/) (47) in order to evaluate if the orthologs of *artA*, *hvpssA*, and *hvpssD* are genomically clustered in other haloarchaea. According to the inspection of more than 70 haloarchaeal genomes, the genes for orthologs of *artA*, *hvpssA*, and *hvpssD* are not clustered.
