# Supplementary figures and images for: Lipid Anchoring of Archaeosortase Substrates and Midcell Growth in Haloarchaea
Source: mBio. 2020 Mar 24;11(2):e00349-20. doi: 10.1128/mBio.00349-20 (PMC7157517; doi:10.1128/mBio.00349-20)

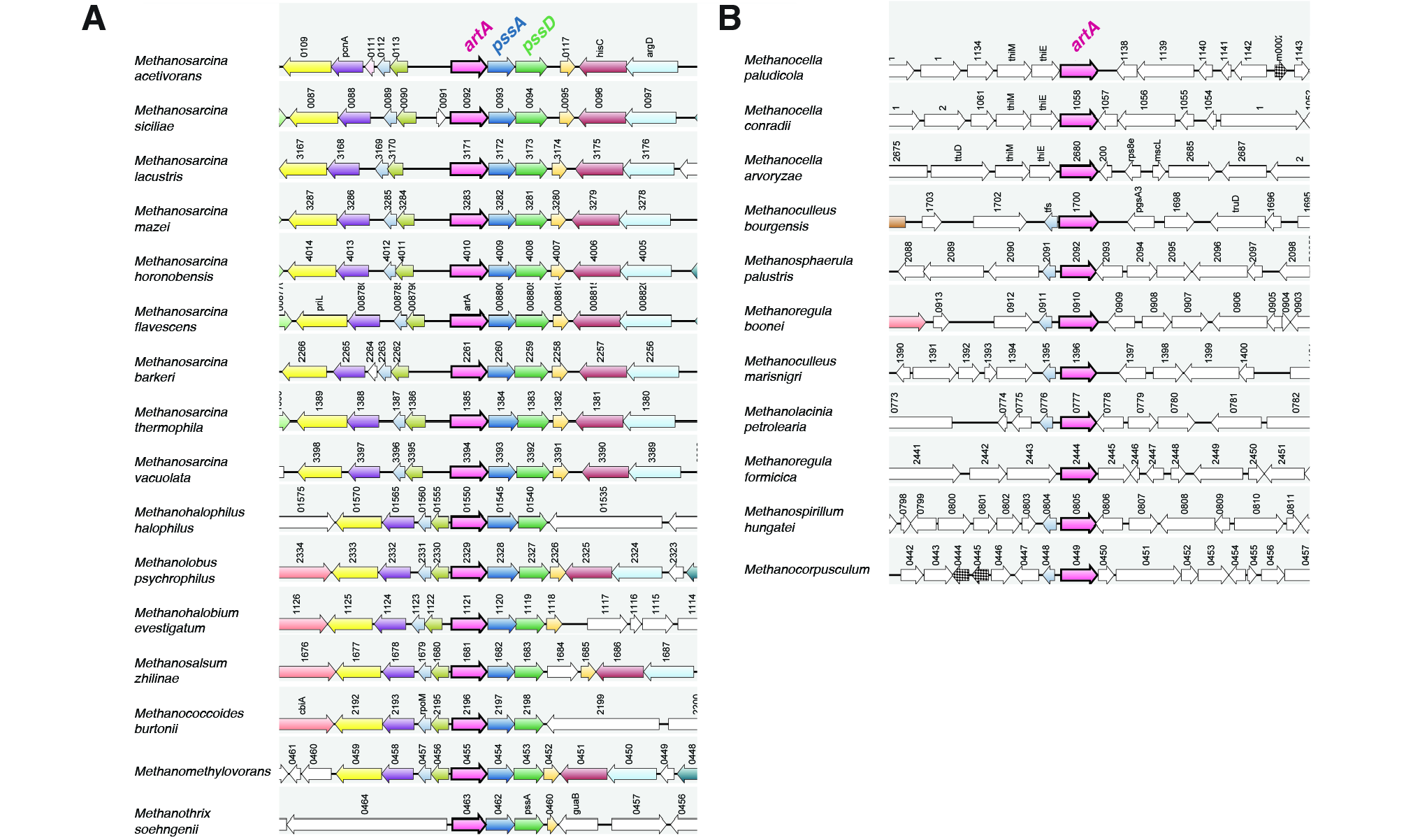

Supplement: FIG S1 [file mBio.00349-20-sf001.tif]

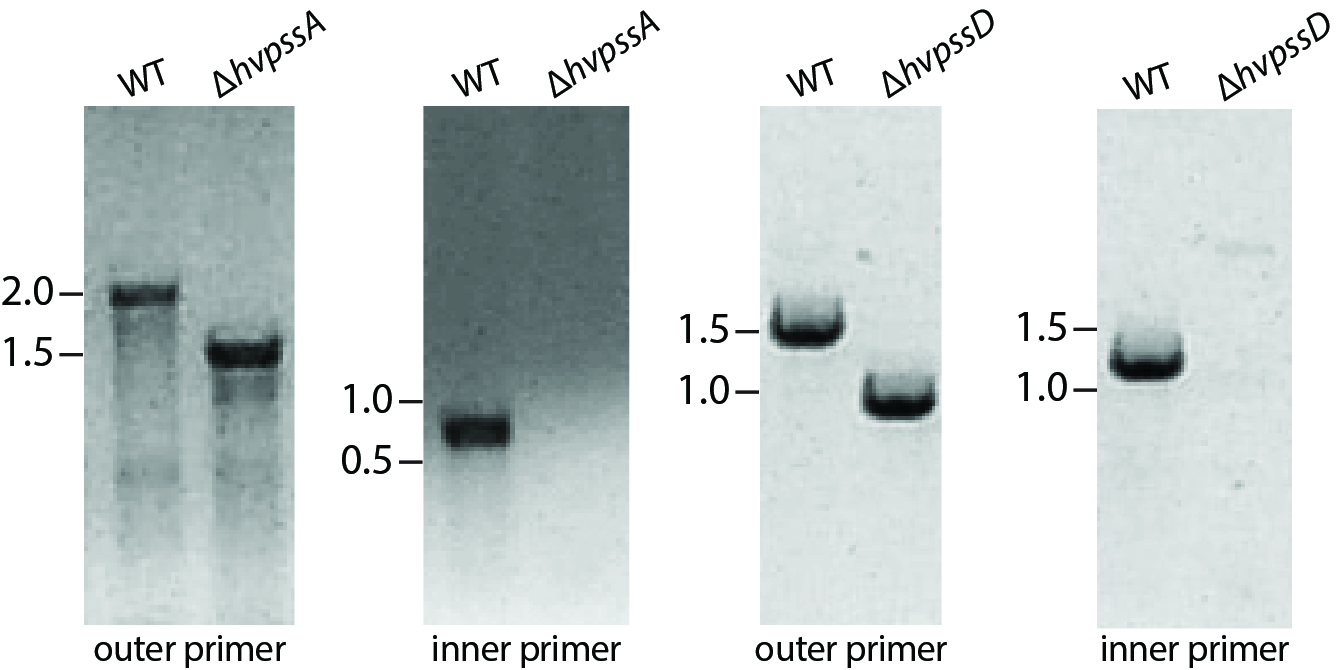

Supplement: FIG S2 [file mBio.00349-20-sf002.tif]

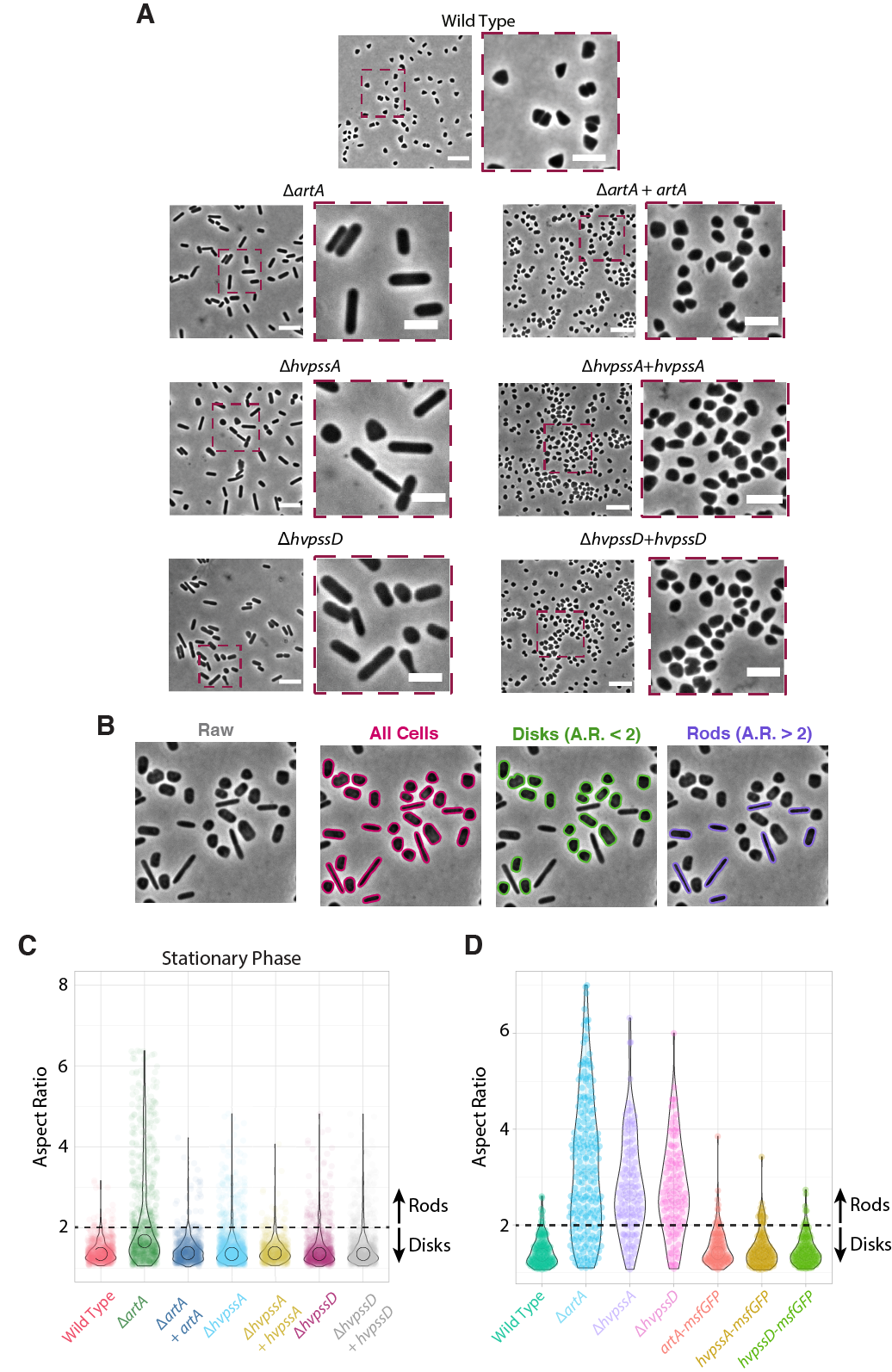

Supplement: FIG S3 [file mBio.00349-20-sf003.tif]

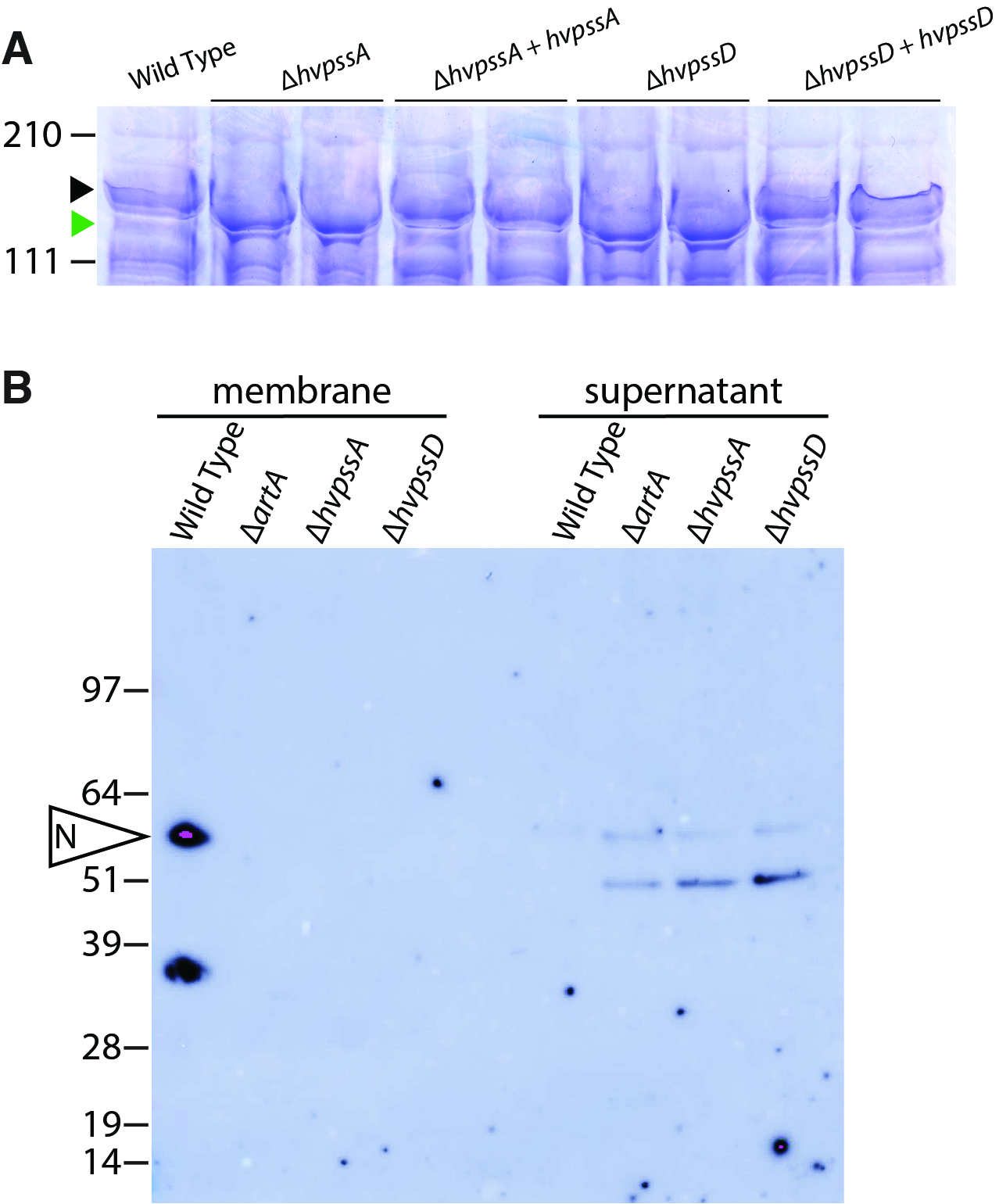

Supplement: FIG S4 [file mBio.00349-20-sf004.tif]

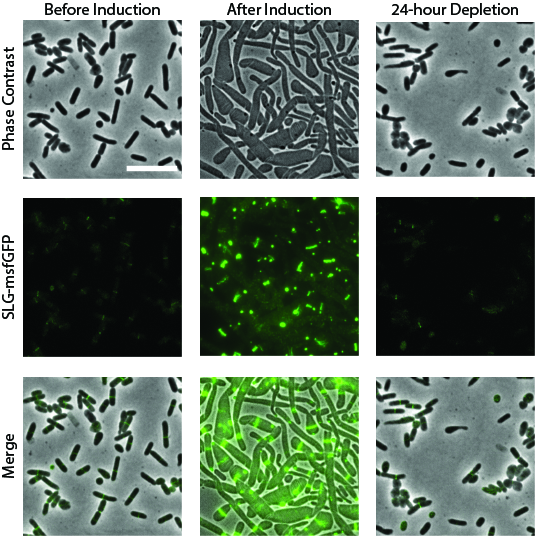

Supplement: FIG S5 [file mBio.00349-20-sf005.tif]
