# Supplementary material for: Lipid Anchoring of Archaeosortase Substrates and Midcell Growth in Haloarchaea
Source: mBio. 2020 Mar 24;11(2):e00349-20. doi: 10.1128/mBio.00349-20 (PMC7157517; doi:10.1128/mBio.00349-20)
Supplement: TABLE S1 [file mBio.00349-20-st001.docx]

**Table S1: Oligos used in this work**

| **Alias** | **5’→3’ Sequence** | **Source** |
| --- | --- | --- |
| FW_pssA_OE_NdeI (IP) | ATTAATCATATGATGAGACCCCGATTCG | This work |
| RV_pssA_OE_EcoRI_His (IP) | TATATTGAATTCTCAGTGATGGTGATGGTGATGGTGATGGTGATGCGGGCCGCCCGCCCAGTAGAAGCG | This work |
| FW_pssA_KO_XbaI (OP) | AATAAAATCTAGAGGCTTTTTATGCGACCGCGCTTCGAGC | This work |
| RV_pssA_KO_XhoI (OP) | AATAAAACTCGAGCCGCGAGCCACGCGTGGTACG | This work |
| RV_pssA_up | CCGTTCCGCCGCCCCGGACCGATGCGATTGCGTACCTGCCC | This work |
| FW_pssA_dw | GGGCAGGTACGCAATCGCATCGGTCCGGGGCGGCGGAACGG | This work |
| FW_pssD_KO_XbaI (OP) | TATATTTCTAGAGTCCGCCGGCTACGC | This work |
| RV_pssD_KO_XhoI (OP) | TATATTCTCGAGCCGTACAGGTCGGCGTAG | This work |
| RV_pssD_up | AGACGGCCAACGCCGACAGCGACGCTTATGCCTCGCCGCGGACGTC | This work |
| FW_pssD_dw | GACGTCCGCGGCGAGGATAAGCGTCGCTGTCGGCGTTGGCCGTCT | This work |
| FW_pssD_OE_NdeI (IP) | TATATTCATATGATGCGGTTCGCACCC | This work |
| RV_pssD_OE_EcoRI_His (IP) | TATATTGAATTCTCAGTGATGGTGATGGTGATGCTCCCGCCGCGCCAA | This work |
| FW_0405_NdeI | TATATTCATATGATGGACCGCCGCCAGTT | This work |
| RV_0405_LVIVD_His_EcoRI | TATATTGAATTCTCAGTGATGGTGATGGTGATGCGGGCCGCCACTCCGATGCTTCGA | This work |
| pTA963-1F | CACACACCAGTCCACGAG | This work |
| pTA963-1R | CGCAATTAACCCTCACTAAAG | This work |
| oHV3 | CGTCCTCCGTAAACCG | This work |
| oHV4 | GTCCGCTACCCTCAAGCTCGACGTAGTCGATGTCT | This work |
| oHV6 | TTAGCCGTCGGCGTC | This work |
| oHV7 | CGTGGATAAAACCCCTCG | This work |
| oHV8 | CGAGGGGTTTTATCCACGTCGAGCCGTCCC | This work |
| oHV9 | CGAAGAAACGGTTTTGTGG | This work |
| oHV81 | CTCGAGGGATCTGGC | This work |
| oHV82 | CCCACTGCCTTGACC | This work |
| oHV83 | GGTCAAGGCAGTGGGGAGCGTGGAAACCTCG | This work |
| oHV101 | CGGACCTATTGCGCATATGCGAAAAGGGGAAGAATTGTTTAC | This work |
| oHV126 | GCTCAAGGAGTCCGC | This work |
| oHV127 | GTCCGCTACCCTCAAGCTCCCGCCGCG | This work |
| oHV128 | CGAGGGGTTTTATCCACGGCGTCGCTGTCGG | This work |
| oHV129 | GCAGGTGTCGATTGCC | This work |
| oHV156 | CGACGACGATGGTGC | This work |
| oHV157 | GTCCGCTACCCTCAAGCGCCCAGTAGAAGCG | This work |
| oHV158 | CGAGGGGTTTTATCCACGCCGGGGCGGC | This work |
| oHV159 | CCGAACTCCGCTTCG | This work |
| oHM6 | CGCCGACGGCTAATCATTTGTAAAGTTCATCCATTCCATGC | This work |
| oHM34 | CTTGAGGGTAGCGGAC | This work |
| oHM68 | GGTGCGGCCGCTCATTTGTAAAGTTCATCCATTCCA | This work |
| oHM91 | AGTACGTATGCCCGGT | This work |
| oHM92 | CGAGTTAGGGCTCGACCTTGAGGGTAGCGGAC | This work |
| oHM93 | CGAGGGGTTTTATCCACGTTACGCCGTCGGAGT | This work |
| oHM94 | CCACATGTTCAGCATATCGG | This work |
| oAB500 | CGGACCTATTGCGCATATGACAAAGCTCAA | This work |
| oAB501 | GCCAGATCCCTCGAGCGCGGCGGCACTTCC | This work |
| oAB502 | CAGAGGTGCGGCCGCTTAGTTCTCGCGGCG | This work |
